# Developing Sustainable Agriculture Systems in Medicinal and Aromatic Plant Production by Using Chitosan and Chitin-Based Biostimulants

**DOI:** 10.3390/plants12132469

**Published:** 2023-06-28

**Authors:** Wenli Sun, Mohamad Hesam Shahrajabian, Spyridon A. Petropoulos, Nazanin Shahrajabian

**Affiliations:** 1 Biotechnology Research Institute, Chinese Academy of Agricultural Sciences, Beijing 100081, China; hesamshahrajabian@gmail.com; 2 Department of Agriculture, Crop Production and Rural Environment, University of Thessaly, 38446 Volos, Greece; 3 Department of Economics, Isfahan (Khorasgan) Branch, Islamic Azad University, Isfahan 81595-158, Iran; nazanin.rajabian@gmail.com

**Keywords:** natural products, chitin, chitosan, medicinal and aromatic plants, biostimulant, stress resistance

## Abstract

Chitosan is illustrated in research as a stimulant of plant tolerance and resistance that promotes natural defense mechanisms against biotic and abiotic stressors, and its use may lessen the amount of agrochemicals utilized in agriculture. Recent literature reports indicate the high efficacy of soil or foliar usage of chitin and chitosan in the promotion of plant growth and the induction of secondary metabolites biosynthesis in various species, such as *Artemisia annua*, *Curcuma longa*, *Dracocephalum kotschyi*, *Catharanthus roseus*, *Fragaria* × *ananassa*, *Ginkgo biloba*, *Iberis amara*, *Isatis tinctoria*, *Melissa officinalis*, *Mentha piperita*, *Ocimum basilicum*, *Origanum vulgare* ssp. *Hirtum*, *Psammosilene tunicoides*, *Salvia officinalis*, *Satureja isophylla*, *Stevia rebaudiana*, and *Sylibum marianum*, among others. This work focuses on the outstanding scientific contributions to the field of the production and quality of aromatic and medicinal plants, based on the different functions of chitosan and chitin in sustainable crop production. The application of chitosan can lead to increased medicinal plant production and protects plants against harmful microorganisms. The effectiveness of chitin and chitosan is also due to the low concentration required, low cost, and environmental safety. On the basis of showing such considerable characteristics, there is increasing attention on the application of chitin and chitosan biopolymers in horticulture and agriculture productions.

## 1. Introduction

Chitin is a linear cationic heteropolymer consisting of *β*-(1-4)-linked 2-acetamde-2-deoxy-D-glucopyranose (N-acetyl-D-glucosamine, GlcNAc) and randomly distributed units of 2-amino-2-deoxy-D-glucopyranose (D-glucosamine, GlcN) [1]. Chitin can be converted to chitosan by both biological and chemical or enzymatic procedures [1]. Chitin is generally available as a structural factor in the exoskeletons of an extensive range of eukaryotic organisms, such as insects; in the cell walls of fungi; in crustaceans, such as crabs, shrimps, and lobsters; and it is the second most plentiful biopolymer on the planet, alongside cellulose [2,3,4,5,6,7,8]. As a natural amino polysaccharide, it indicates different practical applications, owing to its functional characteristics [9,10]. Both chitooligosaccharides and chitosan are obtained from the depolymerization and deacetylation of chitin, respectively [11,12]. Chitin exists naturally in three polymorphic structures: α-, β-, and γ-chitin [13]. A-chitin has anti-parallel chains, whereas β-chitin has parallel chains forming monoclinic crystals with intramolecular H-bonds and intermolecular H-bonds [14]. Li et al. [15] reported that a chitin/cellulose nanofiber complex (CCNFC), which was extracted from the spent mushroom substrate, indicated significant plant growth promotion activity and plant disease resistance activity. It has been reported that chitin-based nanofibers (CNFs) were outstanding in decreasing pathogenic fungal spread in pepper [16,17]. Parada et al. [18] noted that chitin nanofiber may cause a decrease in the number of spots caused by *Alternaria brassicicola* and the size of lesions induced by *Colletotrichum fructicola*, while it can promote disease resistance in strawberry and cabbage plants. Chitin and chitosan are derived from different sources: (1) aquatic: crabs (*Chionoecetes opilio*, *Podophthalmus vigil*, *Paralithodes camtschaticus*, *Carcinus mediterraneus*), water lobster, prawn, krill (*Anax imperator*, *Daphnia longispina*, *Notonecta glauca*, *Hydrophilus piceus*, *Asellus aquaticus*, *Agabus bipustulatus*), and squid pens; (2) terrestrial: spiders (*Geolycosa vultuosa*, *Hogna radiata*, *Nephila edulis*), scorpions (*Mesobuthus gibbosus*), beetles (*Bombyx mori*, *Holotrichia parallela*, *Leptinotarsa decemlineata*), cockroaches, and brachiopods; and (3) microorganisms.

**Figure 1:**
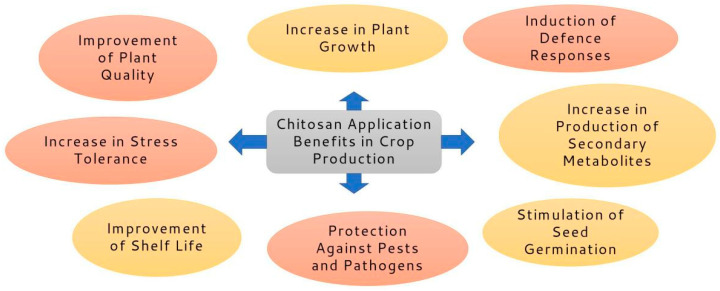
Utilization of chitosan and its derivatives in crop production.

Chitosan and its oligosaccharides may increase photosynthesis by increasing the activities of different enzymes of nitrogen and carbon metabolism, as well as dark and light responses of photosynthetic apparatus. They also have an important function in photosynthetic machinery stimulation by modulating principal photochemistry; they can also manage the restraints of stomata, escalate the carbon fixation effectiveness in dark reactions, and improve the synthesis of metabolites. Chitosan and its oligosaccharides may also promote the biosynthesis of secondary metabolites and the activity of enzymes [19]. Chitosan derivatives have superior antifungal activity compared to chitosan [20]. Chitosan can be used for different purposes based on the specificity of its physicochemical properties [21,22], as well as its biodegradability [23] and biocompatibility [24,25,26]. The potential utilization of chitosan and its derivatives include their use as antimicrobial and antioxidant agents, a drug delivery system, gene therapy, food technology, bio-nanotechnology, regenerative technology, effluent treatments, and electrolytes, as well as in various industries, including paper manufacturing, cosmetics, as absorption enhancers, photography, wood industries, environmental protection, agriculture, obesity treatment, catalyst research, immune therapy, as permeation enhancers, energy production, and in the immobilization of cells. Chitosan leads to various defensive genes in plants, such as genes related to pathogenesis, namely, *chitinase* and *glucanase* [27]. It may also trigger several enzymes in the reactive oxygen species scavenging system, such as superoxide dismutase, peroxidase, and catalase [27,28,29,30]. Chitosan also leads to the accumulation of auxin in the apex of roots, thus promoting root elongation [31]. Moreover, chitosan and its oligosaccharides act as the suited ligand for the initiation of accessible receptors and, therefore, elicit different signaling pathways in the plant, namely, GPCR and PLC/PK, MAPK, H_2_O_2_ burst, and the promotion of transcription parameters, thus generating plant responses to environmental stimuli [32]. Chitosan and its oligosaccharides also show antimicrobial properties and can be used as biopesticides, preventing the proliferation of pathogens and improving crop quality and yield [32,33,34,35]. For example, chitosan has innate antibacterial characteristics that prevent the development of fungi and germs [36]; its nanoparticles were utilized to control the release of citronella essential oil for use against cotton leafworm (*Spodoptera littoralis* (Boisd.) [37]. Moreover, it can noticeably decrease plant oxidative stress and improve crop yield [38,39], while root exposure to chitosan increased both antioxidant and defense enzyme activity [38]. Chitosan can decrease plant diseases via two major procedures: direct antimicrobial function against pathogens, or through the induction of plant defense reactions [39,40,41,42,43]. The effects of chitosan on plant defense mechanisms are connected to its application method and dose [44]. The potential applications of chitosan as eco-friendly tools in agricultural production are shown in Figure 1.

Oligochitosan, obtained from chitosan, is a potential plant immunity regulator [45,46]. It has shown an extensive range of biological applications, including as a plant growth stimulator, antimicrobial agent, and feed additive, among others [47,48]. Oligochitosan can activate a plant’s innate immunity via signal transduction, signal perception, and the oligochitosan response to proteins and genes, thus leading to accumulation of defense-related secondary metabolites [49,50]. It can elicit different defense responses, including the scavenging of reactive oxygen species, the synthesis of jasmonate, the increase of cell walls’ lignification, the production of phytoalexins, and the accumulation of defense-related proteinases inhibitors, as well as the induction of pathogenesis-related (PR) proteins and salicylic acid [51,52,53,54]. Zhang et al. [55] reported that oligochitosan could decrease black spots on tomato fruits, while Deng et al. [56] suggested that a preharvest application of oligochitosan can be an important substitute to conventional control practices for the prevention of post-harvest anthracnose in navel oranges. Oligochitosan showed a significant impact on controlling fruit brown rot; it could boost antioxidant and defense-related enzymes activities, accelerate the corresponding gene transcript expression, and also delay fruit senescence and softening [57]. Oligochitosan remarkably activated phenylpropanoid pathway metabolism and induced defense responses against green and blue mold in citrus fruit [58,59]. Wang et al. [60] proposed that primary metabolites induced by oligochitosan behave as the source of energy and signaling or as substrate molecules that have indirect or direct roles in defense reactions against *Geotrichum candidum*, also known as sour rot, in citrus fruit. 

Relevant literature was obtained using the keywords “Biostimulants”, “Chitin”, “Chitosan”, “Oligochitosan”, “Medicinal plants”, “Aromatic plants”, “Natural products”, “Biotic stress”, and “Abiotic stress” from scientific literature databases, such as “Web of Science”, “SciFinder”, “PubMed”, and “Elsevier”. The current review gives a literature overview with regards to the impacts of chitosan, chitin, and its derivatives on medicinal and aromatic plants, focusing on their noticeable functions in modern sustainable production. We also highlight the major restrictions and illustrate the future possibilities of applications of this form of biostimulant.

## 2. The Effects of Biostimulants on Medicinal and Aromatic Plants

A plant biostimulant is defined as any substance or microorganism used on plants that may lead to increased nutrition efficiency and improvement of abiotic stress tolerance and crop quality traits. Biostimulants consist of algae or plant extracts, mushrooms, amino acids, humic substances, microorganisms, biomolecules, natural ingredients, and metabolites of fermentation, among others [61,62,63,64]. Utilization of Moringa leaf extract led to greater growth, lower weed infestation, and an increased final yield of basil (*Ocimum basilicum* L.) [65,66], while the application of salicylic increased the content of different metabolite components in leaves [67] and improved the values of shoot fresh weight, plant height, and dry weight of basil seedlings under water shortage treatments [68]. The combination of *Azotobacter chroococcum* and *Azospirillum lipoferum* significantly enhanced the fresh and dry yield of coriander [69], while foliar spraying of a biostimulant (Asahi SL or Goemar Goto Arysta Life Science) induced the photochemical effectiveness of photosystem II, stomatal conductance, and transpiration rate, but decreased the intercellular carbon dioxide concentration [70]; moreover, it promoted the total phenolic compounds’ concentration, L-ascorbic acid, and the total antioxidant activity of coriander seedlings [70]. The utilization of different biostimulants as a seed treatment during germination boosted the germination percentage of garden cress (*Lepidium sativum* L.) [71]. Humic acids increased the bulb yield and clover and bulb diameter of garlic (*Allium sativum* L.) [72], and demonstrated a positive effect on the mineral nutrition content [73]. *Bacillus subtilis* and humic acids boosted the level of protein and minerals and increased the antioxidant activities of garlic [74]. Salicylic acid improved the secondary metabolites in a callus culture of Iranian sodab (*Ruta graveolens* L.) [75]. The application of salicylic acid led to a significant decrease of the toxic impact of mercury on the growth and final yield of lemon balm (*Melissa officinalis* L.) [76], while seed priming with *Pseudomonas* spp. accelerated the germination and inoculation of seedlings and positively influenced lemon balm growth [77]. Potassium silicate (K_2_SiO_3_) application increased the pigments, leucine, isoleucine, phenylalanine, ascorbic acid, valine, protein, Ca, Mg, and K content and the uptake of P, K, and N of horseradish tree (*Moringa oleifera* Lam.) [78]. Some other biostimulants, such as benzoic acid, phenylalanine, *N*-benzoyl glycine, glycine, and serine, significantly improved the taxol accumulation in a callus of Japanese yew [79]. The application of humic acids containing L-ascorbic acid and thiamine, both alone (ROOTS) and together with fertilizer (ROOT PLUS), increased the production of marigold (*Calendula officinalis* L.) plants; it also induced earlier flowering [80]. For the same species, bioactive amino acid constituents, namely Kadostim and Humiforte, promoted the total flavonoids and carbohydrate constituents of leaves, P, K, and N [81]. The application of Radifarm^®^ boosted the nutrient status of French marigold (*Tagetes erecta* and *Tagetes patula* L.) plants and flowering [82]. The utilization of chitosan reduced the adverse effects of salt stress, and stimulated the enzyme activities and antioxidants of milk thistle (*Silybum marianum* L.) [83]. Salicylic acid decreased the osmotic adjustment under drought stress, increased the antioxidant compounds’ content in seeds, and boosted the vegetative growth and yield of milk thistle by escalating the relative water content in regions under the influence of a water shortage [84]. *Trichoderma ovalisporum* (NFCCI2689) and *T. harzianum* (NFCCI 2241) improved the menthol content and oil yield of mint (*Mentha arvensis* L.) [85], while the application of CRADLE^TM^, Mobilizer^TM^, and Nanozim NXT^TM^ biostimulants promoted metabolic and physiological responses, such as the leaf water potential, gas exchange, proline accumulation, and relative water content of stressed seedlings of mint [86]. The application of ethanol and humic acids improved the photosynthetic pigments and yield potential of Moldavian dragonhead (*Dracocephalum moldavica* L.) [87]. The application of salicylic acid stimulated the oil components of peppermint (*Mentha piperita* L.) compared to control plants, particularly menthol, menthone, methyl acetate, and 1,8-cineole [88]. Applications of chitosan, citric acid, and humic acid on the same species boosted the stem and leaves dry weight [89,90]. A combined application of arbuscular mycorrhizal fungi with chitosan positively affected the secondary metabolites and dry herbal weight of peppermint [91,92]. Moreover, *Pseudomonas* sp. P17 strain was used as a potential plant growth promoter for better plant growth and higher nutrient uptake in pigeon pea (*Cajanus cajan* L.) [93]. A chitosan and salicylic acid application stimulated the production of amarogentin, mangiferin, and swertiamarin from shoot cultures of swertia (*Swertia paniculata* Wallich), which have significant bioactive properties that render them very important in the pharmaceutical industries [94].

## 3. Practical Usage of Chitosan on Medicinal and Aromatic Plants

The multiple functions of chitosan’s chemically and naturally synthesized components could act as potent elicitors and biostimulants and change the physicochemical status of medicinal crops [95,96,97]. They participate in the activation of defensive genes via the overexpression of pathogenesis-related genes; they may also provide photoprotection by increasing secondary metabolite biosynthesis. They play an important function in oxidative damage reversal by managing hydrogen peroxide and nitric oxide signaling. They also stimulate activity at the cellular level of antioxidant enzymes, such as peroxidase, dismutase superoxide, and catalase; retain ion homeostasis; improve the photosynthesis rate by interacting with abscisic acid-induced stomatal closure; improve plant vigor and environmental stress tolerance; and generally improve crop performance [98,99,100]. Aristolochic acid I (AA I) is one of the substituted 1-phaenanthrene carboxylic acids usually discovered in *Asarum* and *Aristolochia* plants, and chitosan-changed carbon microcoils were used as suitable adsorbents for the selective extraction of aristolochic acid I from Aristolochiaceae plants [101]. *Psammosilene tunicoides* W. C. Wu et C. Y. Wu, a monotypic species of Caryophyllaceae that is endemic to China, has been used as folk medicine for centuries, and it is one of the major ingredients in the traditional Chinese medicine “Yunnan Baiyao”. According to the literature, hairy roots of the species elicited with 200 mg L^−1^ of chitosan increased the total saponin content by 4.55-fold [102]. High-performance liquid chromatography (HPLC) indicated that the yields of gypsogenin, quillaic acid, and gypsogenin-3-O-*β*-D-glucuronopyranoside were significantly escalated after a chitosan application [102]. Furthermore, exogenous chitosan usage significantly triggered the reactive oxygen species (ROS), scavenging enzyme activities, and nitric oxide (NO) content in the hairy roots of *Psammosilene tunicoides* [102]. 

In basil seedlings, the level of rosmarinic acid (RA) increased after the application of 100 mg/L of chitosan lactate [103]. The mixture of common chili (*Capsicum annuum*) fruit extracts and chitosan can be effective against pathogenic bacteria, especially against *Staphylococcus aureus*, which is a Gram-positive, spherically shaped bacterium [104]. A chitosan application increased the content of the EO components, the positive impacts of chitosan utilization under drought stress conditions [105]. The usage of water-soluble carboxymethyl chitosan-grafted daphnetin nanoparticles (DA@CMCS-NPs) improved the activities of defense enzymes in tobacco, and effectively inhibited the development of tobacco bacterial wilt (*Ralstonia solanacearum*) [106]. The effects of chitosan on different medicinal and aromatic plants are shown in Table 1. 

## 4. Activities and Applications of Oligochitosan

Chitooligosaccharides or oligochitosan are plant elicitors that have similar impacts to chitosan on herbs and plants against biotic stress [136]. Moreover, oligochitosan, with a higher degree of polymerization, indicated a stronger elicitation impact via the overexpression of defensive genes [137]. Oligochitosan may promote protection against pathogens through the activation of salicylic acid (SA) or jasmonic acid/ethylene (JA/ET)-dependent pathways; boost growth by escalating gibberellin, auxin, photosynthesis content, and N and C assimilation; induce protection against abiotic stress via the abscisic acid (ABA)-dependent pathway; and increase the synthesis of secondary metabolites with medicinal and antipathogenic activities [138]. The application of chitosan oligosaccharide at both 60 g L^−1^ and 120 g L^−1^ levels increased fennel growth and yield, while it increased estragole and reduced the anethole content, thus demonstrating adverse effects on the essential oil quality of fennel [139]. Liu et al. [140] reported that oligochitosan sprayed at 5 g/L noticeably inhibited rhizome rot caused by *Fusarium oxysporum*, and also decreased weight loss; however, it did not influence the amount of soluble solids or titratable acidity of the rhizomes of ginger (*Zingiber officinale* Roscoe). The potential of oligochitosan to decrease rot in stored rhizomes could be related to their ability to induce defense responses in ginger [140]. Li et al. [141] also indicated that oligochitosan caused the accumulation of reactive oxygen species, mitochondrial dysfunction, metacaspase activation, and Ca^2+^ homeostasis dysregulation, together with hallmarks of apoptosis consisting of DNA fragmentation, phosphatidylserine externalization, and nuclear condensation to control pathogens in *Ceratocystis fimbriata*. Lu et al. [142] observed that oligochitosan led to tobacco resistance to tobacco mosaic virus (TMV) by the Ca^2+^ signaling pathway. Ahmad et al. [143] indicated that a foliar spray of irradiated chitosan (80 mgL^−1^) significantly increased the physiology and growth of peppermint. On the basis of LC-MS analysis, the chitosan oligosaccharide application slightly increased artemisinin production in the leaves, while the impacts of chitosan oligosaccharide and salicylic acid on artemisinin production varied, indicating that these elicitors may not be an appropriate method for increasing the artemisinin yield or that the regulation takes longer to be effective in sweet wormwood (*Artemisia annua* L.) [144]. 

## 5. The Application of Chitin as Biostimulant

Chitin can induce both a local immune reaction and a systemic disease resistance when provided as a supplement in soils [145]. The most important chitin derivatives are chitosan, alkyl chitin, N and O sulfated chitin, dibutyryl chitin, carboxymethyl chitin (CMCH), chito-oligosaccharides (COS), chitin nanofibers (CNF), chitin nano-whiskers (CNW), chitin nanoparticles (CNP), chitin nanocomposites (CNC), and chitin hydrogels (CHG). Unlike chitosan, the application of chitin had no remarkable effects on defense mechanisms, such as enzymatic activities, gene expression levels, hormone quantities, and root secreted proteins of medicinal cannabis (*Cannabis sativa* L.) [146,147,148,149]. Cretoiu et al. [150] added chitinous matter obtained from shrimps to the soil’s top layer to assess the suppressiveness of the oil toward *Verticillium dahliae*, as well as against plant pathogenic nematodes. They reported a change in both structures and an abundance of soil microbial communities, both total soil fungi and bacteria; they also found that the structures and abundance of soil actinobacteria and the Oxalobacteraceae were influenced by chitin. Liopa-Tsakalidi et al. [151] reported that a chitin application in the peat substrate did not influence the weight and length of lemon balm. Chitin influenced the tarragon leaves, leading to an increase of the total chlorophyll content [151]. The seed germination percentage of chervil (*Anthriscus cerefolium* L.) was improved in the substrate with 1% and 2% chitin and 200, 500, and 1000 ppm GA_3_ compared to the control treatments (H_2_O) [152]. With the increasing rate of chitin and GA_3_, the seed germination of the chervil was decreased (Liopa-Tsakalidi and Barouchas, 2011). In the combinations of 1% chitin + 80 mM NaCl, 1% chitin + 120 NaCl, 120 NaCl + GA_3_, 2% chitin + 180 NaCl, and 200 GA_3_ +180 NaCl, germination was boosted and the Timson index of germination velocity was greater than the corresponding velocity in H_2_O [152]. Kanawi et al. [153] found that the percentage of germination of basil seeds increased with the increasing concentration of chitin, while the percentage of inhibition of pathogenic fungi also escalated with the rise of the concentration of chitin. The application of chitin/nano-TiO_2_ and chitin/nano-SiO_2_ resulted in a notable increase of the preservation of seed quality of Ginkgo (*Ginkgo biloba* L.) [154].

## 6. Conclusions—Future Remarks

Biostimulants are known and described as molecules of biological origin with positive effects on the growth and development of different crops, increasing resistance to both biotic and abiotic stress parameters and improving crop quality and yield. The most important biostimulant impacts on crops are improved stress tolerance, the production of deeper roots, increased cation exchange, accelerated crop establishment, improved seed germination rates, reduced leaching, increased nutrient uptake, detoxification of heavy metals, increased stomata opening and plant transpiration, improved drought tolerance, increased natural plant toxins, repelling of pests, stimulation of immune system of plants, and improved yields and crop performance. The accurate application of chitin may show a beneficial function in the growth, development, and bioactive phytoconstituents accumulation of medicinal plants, which, in turn, could upscale the possibility of industrial exploitation of medicinal plants in the most sustainable way possible. Chitosan is the principal tool in the agricultural field because it is biocompatible, nontoxic, and biodegradable, and also has adsorption capabilities, since it can absorb different heavy metals, insecticides from water or pollutants, soil, and pesticides. Chitosan can be formed in several ways, as nanoparticles, nanomaterials, and nanocomposites, which are appropriate for plant protection, while its characteristics can be improved by combining it with other substances. The importance of chitosan in the formation of photosynthetic metabolites and pigments; the absorption of elements; assimilation; and the expression of genes as micro-, macro-, and nano-structures and in the form of composites or pure components with metal elements; and, lastly, the qualitative and quantitative performance of different medicinal plants and crops influenced by chitosan components has been investigated thus far. Chitosan and chitosan-based nanoparticles have shown promising findings in medicinal plants as fertilizers, plant disease control and insecticidal agents, soil-conditioning agents, ripening retardants, and fruit and seed coatings. These components are able to increase the assimilation of secondary metabolites through the stimulation of biochemical modification, defensive responses, and the biosynthesis of phytoalexins. Using chitosan as a foliar spray significantly increased plant growth; ions; chlorophyll; antioxidant capacity; and phytopharmaceutical components, such as anthocyanins, total soluble phenols and flavonoids, and alkaloids; it also reduced the number of oxidative biomarkers. Biocompatible and biodegradable chitosan and chitosan-based nanomaterials are becoming important in agriculture because of their unique characteristics, such as eliciting, biostimulating, antimicrobial activity, and stimulation of plant growth and tolerance to environmental stresses. Currently, these substances are not being commonly utilized in agriculture, as the procedures of their biological activity in plants and action against pathogenic microorganisms have not been completely elucidated to date. Chitosan successfully increases the physiological functions of plants; additionally, chitosan treatment modulates various genes, especially through the activation of plant defense signaling pathways. Furthermore, it is useful in decreasing fertilizer losses because of its coating ability, which is essential in maintaining control of environmental pollution. Chitin and its derivatives are highly recognized as growth stimulants, plant growth regulators, and elicitors for the production of secondary metabolites. In summary, despite the open challenges, molecules of chitosan and chitin have been proven practical in different aspects of plant biology, from the increase in crop yield to the protection against attacks from pathogens. Hence, more research is needed to survey and fine tune the application of chitosan and chitin to the root zone to prevent soil-borne diseases and to decrease the overuse of synthetic fertilizers in medicinal and aromatic crops in the future. 

## Figures and Tables

**Table 1:** The effects of chitosan on medicinal and aromatic plants.

Plant	Scientific Name	Plant Family	Key Point	References
Basil	*Ocimum basilicum*; *Ocimum ciliatum*	Lamiaceae	Various amounts of chitosan had considerable impacts on the antioxidant activity and total phenol content of the extracts of two species.	[107,108]
			Chitosan could be a promising material used to reduce the adverse impacts of water stress on the growth factors of basil seedlings.	[108]
Bitter melon	*Momordica charantia* L.	Cucurbitaceae	An application of chitosan and their conjugated forms as nanoparticles (Se-CS NPs) improved the biochemical and morphophysiological characteristics of bitter melon plants under moderate and severe salinity stress condition.	[109]
			Exogenous utilization of Se-CS NPs leads to the expression of multiple defense- and secondary metabolism-related transcripts in bitter melon plants.	[109]
*Dendrobium* orchids	*Dendrobium*	Orchidaceae	Chitosan showed the ability to improve floral production of the *Dendrobium*.	[110]
			It also stimulated the number of vascular bundles in both young and old leaves.	[110]
Dragonhead	*Dracocephalum kotschyi* Boiss	Lamiaceae	It was an effectual elicitor for increasing rosmarinic acid and quercetin content.	[111]
			Chitosan increased the content of apigenin noticeably.	[111]
			A chitosan spray had a significant influence on the principle essential oil components, such as *p*-cymene and thymol.	[112]
Fenugreek	*Trigonella foenum-graecum* L.	Leguminosae	The activity of nitrate reductase and the contents of photosynthetic pigments and carbonic anhydrase enzymes were significantly increased after an application of Co-60 gamma-irradiated chitosan.	[113]
			Co-60 gamma-irradiated chitosan significantly boosted the total alkaloid content, seed yield, and trigonelline constituent.	[113]
Galega	*Galega officinalis* L.	Leguminosae	An application of chitosan had a more optimal impact on the morphological characteristics.	[114]
German chamomile	*Matricaria recutita* L.	Asteraceae	Its foliar spray in the flowering stage increased the chamomile flower number and weight per plant.	[115]
			Its usage at the rate of 40 ppm is suggested for German chamomile cultivation.	[115]
Ginger	*Zingiber officinale* L.	Zingiberaceae	Its application induced the HR marker gene *HSR203J* in ginger and inhibited the expression of cell death markers *AIF2*, *HIN1*, and *AIF1*.	[116]
			Chitosan has the potential to prime standing ginger plants against soft rot disease and facilitate appropriate sustainable management of ginger soft rot (*Pythium myriotylum*).	[116]
Hyssop	*Hyssopus officinalis* L.	Lamiaceae	The results show that spraying chitosan at 2.5 g/L significantly increased the measurements of the canopy diameter, inflorescence height, plant height, numbers of auxiliary and flowering branches, dry herbal weight, and the components of photosynthesis pigments under various levels of irrigation frequencies.	[117]
			The maximum values of the volatile oil yield were related to the chitosan-spraying in a reduced irrigation condition.	[117]
			Chitosan had noticeable effects on the percentage of (*cis*- and *trans*-) pinocamphone, as the predominant components of hyssop volatile oil, under a reduced irrigation condition.	[117]
Indian borage (Indian mint)	*Coleus aromaticus* Benth (L.)	Lamiaceae	A mixture of Chitosan and cytokinin showed a positive impact on multiple shoot induction than cytokinin alone.	[118]
			Its application also increased the percentage of alkaloids, flavonoids, terpenoids, saponins, tannins, and total phenolic content.	[118]
Lavender-cotton	*Santolina chamaecyparissus* L.	Asteraceae	Its application improved antifungal capacities and increased some physical characteristics.	[119]
Lemon balm	*Melissa officinalis* L.	Lamiaceae	It increased plant regeneration in a callus culture of a medicinal herb.	[120]
			Its consumption consists of the trigger of defense-related enzymes; stimulated expression of rosmarinic acid synthase (RAS) genes and tyrosine aminotransferase (TAT); and stimulation of methyl jasmonate biosynthesis.	[121]
Milk thistle	*Silybum marianum* (L.) Gaertn.	Asteraceae	Significant reduction of the negative impact of salinity and increased plant growth and improved physiological characteristics occurred after its application.	[83]
			Its usage at 0.01% increased the chlorophyll and total chlorophyll content.	[83]
			Its application promoted the enzymatic activity and reduced H_2_O_2_ components in the leaves.	[83]
			Chitosan may protect plants from salt stress damage by regulation of the intracellular ion component and through increasing the capability of antioxidant enzyme activities.	[83]
Moringa	*Moringa oleifera* L.	Moringaceae	Chitosan improved the plant growth parameters: root length, root fresh weight, shoot length, shoot fresh weight, shoot dry weight, root dry weight, and contents of chlorophyll-b and chlorophyll-a.	[122]
Peppermint	*Mentha piperita* L.	Lamiaceae	Under drought stress, the usage of chitosan-coated iron oxide nanoparticles (Fe-CTs NPS) increased essential oils production in the mint plant.	[123]
			Under foliar spray of chitosan, the maximum constituents of the essential oil yield, menthol, and the balance of menthol/menthone of the essential oil from peppermint were achieved from the peppermint plants.	[92]
Roselle	*Hibiscus sabdariffa* L.	Malvaceae	Chitosan nanofiber (CNF) treatments affected the plant growth regulators’ impact on most of the traits evaluated.	[124]
			It could be applied for enhancement of the number of calyxes, plant height, total chlorophyll, β-carotene, antioxidant activity, and flavonoids.	[124]
Savory	*Satureja hortensis* L.	Lamiaceae	Under severe water stress, its application improved the total soluble sugar, antioxidant activity, total phenolic and proline contents, and essential oil content of the seedlings.	[125]
			Under water stress conditions, it may have positive influences on the essential oil quality and quantity, osmotic adjustment, antioxidant activity, and growth of summer savory.	[125]
Spearmint	*Mentha spicata* L.	Lamiaceae	Chitosan–melatonin nanoparticles could be used as an innovative protective agent to reduce the impact of salinity in spearmint plants.	[126]
St. John’s wort	*Hypericum perforatum* L.	Clusiaceae	It showed a significant rise in xanthone production and a simultaneous decline in flavonoid production.	[127]
			Chitosan also led to the production of 1,7-dihydroxyxanthone (euxanthone).	[127]
Sweet marjoram	*Majorana hortensis* Moench	Lamiaceae	Its application improved the marjoram biomass and secondary metabolites’ assimilation.	[128]
Tashenehdari	*Scrophularia striata* Boiss.	Scrophulariaceae	The increase in amino acid content and phenylalanine ammonia-lyase (PAL) activity was related to rises in the phenolic components after application of chitosan treatments.	[129]
			Chitosan, by up-regulating the *PAL* gene, increases the production of the phenylpropanoid content.	[129]
Thyme	*Thymus daenensis* Celak	Lamiaceae	Under the mild stress, the maximum essential oil yield was related to the utilization of 400 μL^−1^ chitosan.	[130]
Mediterranean thyme	*Thymbra spicata* L.	Lamiaceae	The carvacrol level in the essential oil escalated in the plants after application of chitosan.	[131]
			The spray of chitosan could reduce the harmful impacts of water shortage on the oil yield and the carvacrol percentage.	[131]
Russian sage	*Salvia abrotanoides* (Kar.) Sytsma	Lamiaceae	A chitosan foliar application can increase drought tolerance.	[132]
			The leaves showed significant antioxidant enzyme activity under drought stress using chitosan nanoparticles.	[132]
Turmeric	*Curcuma longa* L.	Zingiberaceae	Its application improved the production of curcumin.	[133]
			The chitosan could boost the activity or protease inhibitor in rhizomes and leaves.	[133]
Wild mint	*Mentha arvensis* L.	Lamiaceae	The 0.125% content of chitosan provides a higher accumulation of roots, shoots, and total dry weight. The maximum menthol content is obtained after application of 0.06% chitosan.	[134]
Winged-fruited marigold	*Calendula tripterocarpa* Rupr.	Asteraceae	The chitosan application also increased the growth parameters, carotenoids, and levels of chlorophyll *a*, *b* under both Ni stress and normal conditions.	[135]
			Chitosan decreased the level of malondialdehyde and the activities of catalase (CAT) and superoxide dismutase (SOD) in the shoot and roots under Ni stress.	[135]

## Data Availability

Not applicable.
